# Supplementary material for: Structural polymorphism and population-variable coding capacity of HERV-K(HML-2) in human pangenomes
Source: bioRxiv. 2026 Sep 28:2026.09.22.753638. Preprint. [Version 1] doi: 10.64898/2026.09.22.753638 (PMC13648407; doi:10.64898/2026.09.22.753638)
Supplement: Supplementary file 2 [file media-2.zip › Supplementary_Data/Figure_8_population_metadata_sources/coriell_HG06807.html]

HG06807


Coriell Institute for Medical Research

- Request a Quote for Custom Services
- Donate
- Login
- View Cart


Samples
OR

Website

Sample Catalog |
Custom Services |
Core Facilities |
Genomic Data Search

Navigation Header

- Biobank
  - NRGR
  - NIGMS
  - NINDS
  - NIA
  - NHGRI
  - NEI
  - Allen Cell Collection
  - Rett Syndrome iPSC Collection
  - Autism Research Resource
  - HD Community Biorepository
  - CDC Cell and DNA
  - J. Craig Venter Institute
  - Orphan Disease Center Collection
  - All Biobanks
- Research
  - Overview
  - Meet Our Scientists
    - Our Faculty
    - Our Scientific Staff
  - Camden Cancer Research Center
  - Epigenetic Therapies SPORE
  - Core Facilities
  - Epigenomics
  - Camden Opioid Research Initiative (CORI)
  - The Issa Lab
  - The Jian Huang Lab
  - The Luke Chen Lab
    - The Lab
    - The Team
    - Publications
  - The Scheinfeldt Lab
  - The Shumei Song Lab
  - The Nora Engel Lab
    - The Lab
    - The Team
    - Publications
  - Publications
- Services
  - Overview
  - Biobanking Services
    - Core Services
    - Project Management
    - Research Support Services
    - Sample Cataloging
    - Sample Collection Kits
    - Sample Data Management
    - Sample Distribution
    - Sample Management
    - Sample Procurement
    - Sample Storage
  - Bioinformatics and Biostatistics Services
  - Cellular and Molecular Services
    - Biomarker Research Solutions
    - Cell Culture
    - Nucleic Acid Isolation and Quality Control
  - Clinical Trial Support
    - Overview
    - Sample Collection
    - Data Management
    - Sample Processing and QC
    - Storage and Distribution
    - Biomarker Services
    - Data Analaysis
  - Core Facilties
    - Overview
    - Animal and Xenograft
    - Bioinformatics and Biostatistics
    - Cell Imaging
    - CRISPR Gene Engineering
    - Flow Cytometry and Cell Sorting
    - Genomics and Epigenomics
    - iPSC - Induced Pluripotent Stem Cells
    - Organoids
  - Coriell Marketplace
  - Genomic, Epigenomic and Multiomics Services
  - Stem Cells and iPSC Services
    - Core Services
    - Reprogramming
    - Characterization and Quality Control
    - Differentiated Cell Lines
    - iPSC-Derived Organoids
    - iPSC Expansion
    - iPSC Gene Editing
- Ordering
  - Stem Cells
  - Cell Lines
  - DNA and RNA
  - Featured Products
    - FFPE
    - HMW DNA
  - Genomic Data Search
  - Search by Catalog ID
  - Help
    - Create Account
    - Order Online
    - Ordering FAQ
    - FAQs/Culture Instructions
    - Reference Materials
      - Biobanks
      - NIGMS Repository
      - NHGRI Repository
      - NINDS Repository
      - NIA Repository
      - NIST
      - GeT-RM
    - Secondary Distribution Policies
    - MTA Assurance Form
    - Shipment Policy
    - Contact Customer Service
- About Us
  - About Coriell
  - Meet Our Team
  - Meet Our Board
  - Education
    - Science Fair
    - Outreach
    - College Internships
  - Press Room
    - Press Releases
    - Coriell Blog
    - Annual Report
  - Careers
    - Working at Coriell
    - Verifications of Employment
  - Giving
    - Donate
    - Giving FAQ
  - Contact Us
  - Notices
    - Legal Notice
    - IBC Minutes

- Login


  View Cart


search submit

HG06807

**LCL** from **B-Lymphocyte**

#### Description:

#### AFRICAN AMERICANS LIVING IN ST. LOUIS, MISSOURI

#### Affected:

#### No Data

#### Sex:

#### Female

#### Age:

#### No Data

Sample Description

- **Overview**
- **Phenotypic Data**


- **Culture Protocols**


## Overview

|  |  |
| --- | --- |
| **Repository** | NHGRI Sample Repository for Human Genetic Research |
| **Subcollection** | NHGRI Sample Repository for Human Genetic Research |
| **Cell Type** | B-Lymphocyte |
| **Transformant** | Epstein-Barr Virus |
| **Sample Source** | LCL from B-Lymphocyte |
| **Country of Origin** | USA |
| **Family Member** | 2 |
| **Family History** | N |
| **Relation to Proband** | mother |
| **ISCN** | 46,XX[20] |
| **Species** | Homo sapiens |
| **Common Name** | Human |
| **Remarks** | These cell lines and DNA samples were prepared from peripheral blood samples collected from people living in the St. Louis, MO metropolitan area who self-identified as African American. |

## Phenotypic Data

|  |  |
| --- | --- |
| **Remarks** | These cell lines and DNA samples were prepared from peripheral blood samples collected from people living in the St. Louis, MO metropolitan area who self-identified as African American. |

## Culture Protocols

|  |  |
| --- | --- |
| **Split Ratio** | 1:4 |
| **Temperature** | 37 C |
| **Percent CO2** | 5% |
| **Percent O2** | AMBIENT |
| **Medium** | Roswell Park Memorial Institute Medium 1640 with 2mM L-glutamine or equivalent |
| **Serum** | 15% fetal bovine serum Not Inactivated |
| **Substrate** | None specified |
| **Subcultivation Method** | dilution - add fresh medium |
| **Supplement** | - |

Pricing

International/Commercial/For-profit:

$448.00USD

U.S. Academic/Non-profit/Government:

$225.00USD

Add to Cart

How to Order

- Ordering Instructions
- MTA / Assurance Form
- Statement of Research Intent Form

Related Products

Same Subject

- HG06807 - DNA
- HG06807 - Stem cell

Same Family

- 3559

Miscellaneous

- Custom Services

Our mission is to prevent and cure disease through biomedical research.

CONTACT US

Catalog Inquiries

Email  customerservice@coriell.org

Mailing Address

Coriell Institute for Medical Research  
403 Haddon Avenue Camden, NJ 08103, USA  
Phone: (856) 966-7377

Subscribe to our newsletter here

Ⓒ 2026 Coriell Institute. All rights reserved.

- Facebook
- Linkedin

- Youtube
