# Supplementary material for: Structural polymorphism and population-variable coding capacity of HERV-K(HML-2) in human pangenomes
Source: bioRxiv. 2026 Sep 28:2026.09.22.753638. Preprint. [Version 1] doi: 10.64898/2026.09.22.753638 (PMC13648407; doi:10.64898/2026.09.22.753638)
Supplement: Supplementary file 2 [file media-2.zip › Supplementary_Data/Table_S14/phylogeny_review/Figure_3.pdf]

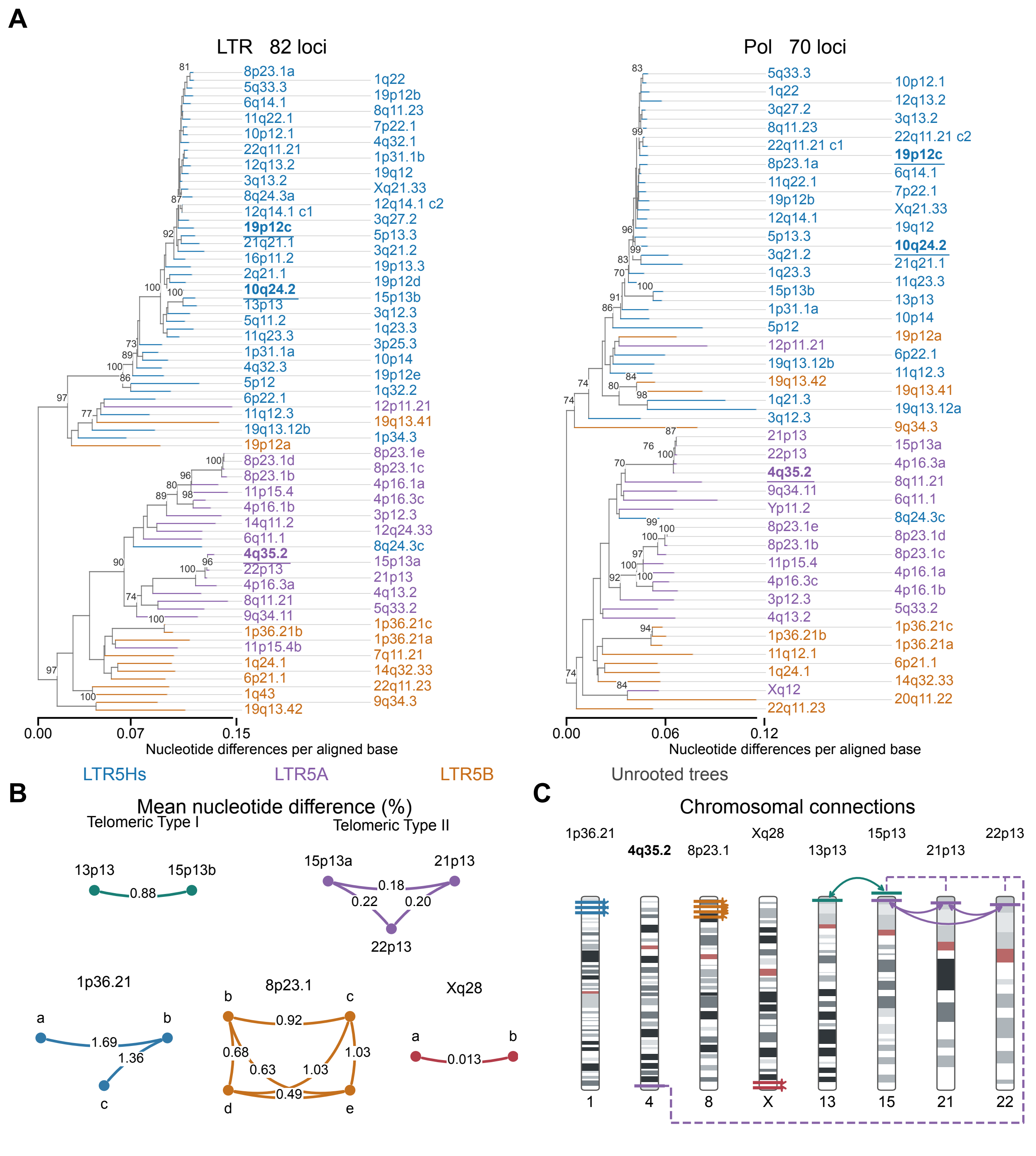
A
LTR 82 loci
8p23.1a
5q33.3
6q14.1
11q22.1
10p12.1
22q11.21
12q13.2
3q13.2
8q24.3a
12q14.1 c1
19p12c
21q21.1
16p11.2
2q21.1
10q24.2
13p13
5q11.2
11q23.3
1p31.1a
4q32.3
5p12
6p22.1
11q12.3
19q13.12b
19p12a
8p23.1d
8p23.1b
11p15.4
4p16.1b
14q11.2
6q11.1
4q35.2
22p13
4p16.3a
8q11.21
9q34.11
1p36.21b
11p15.4b
1q24.1
6p21.1
1q43
19q13.42
1q22
19p12b
8q11.23
7p22.1
4q32.1
1p31.1b
19q12
Xq21.33
12q14.1 c2
3q27.2
5p13.3
3q21.2
19p13.3
19p12d
15p13b
3q12.3
1q23.3
3p25.3
10p14
19p12e
1q32.2
12p11.21
19q13.41
1p34.3
8p23.1e
8p23.1c
4p16.1a
4p16.3c
3p12.3
12q24.33
8q24.3c
15p13a
21p13
4q13.2
5q33.2
1p36.21c
1p36.21a
7q11.21
14q32.33
22q11.23
9q34.3
0.00
0.07
0.15
Nucleotide differences per aligned base
Pol 70 loci
5q33.3
1q22
3q27.2
8q11.23
22q11.21 c1
8p23.1a
11q22.1
19p12b
12q14.1
5p13.3
3q21.2
1q23.3
15p13b
1p31.1a
5p12
12p11.21
19q13.12b
19q13.42
1q21.3
3q12.3
21p13
22p13
4q35.2
9q34.11
Yp11.2
8p23.1e
8p23.1b
11p15.4
4p16.3c
3p12.3
4q13.2
1p36.21b
11q12.1
1q24.1
Xq12
22q11.23
10p12.1
12q13.2
3q13.2
22q11.21 c2
19p12c
6q14.1
7p22.1
Xq21.33
19q12
10q24.2
21q21.1
11q23.3
13p13
10p14
19p12a
6p22.1
11q12.3
19q13.41
19q13.12a
9q34.3
15p13a
4p16.3a
8q11.21
6q11.1
8q24.3c
8p23.1d
8p23.1c
4p16.1a
4p16.1b
5q33.2
1p36.21c
1p36.21a
6p21.1
14q32.33
20q11.22
0.00
0.06
0.12
Nucleotide differences per aligned base
LTR5Hs
LTR5A
LTR5B
Unrooted trees
B
Mean nucleotide difference (%)
Telomeric Type I
13p13
15p13b
0.88
Telomeric Type II
15p13a
21p13
0.18
0.22
0.20
22p13
1p36.21
a
b
1.69
1.36
c
8p23.1
b
c
0.92
0.68
0.63
1.03
1.03
0.49
d
e
Xq28
a
b
0.013
C
Chromosomal connections
1p36.21
4q35.2
8p23.1
Xq28
13p13
15p13
21p13
22p13
1
4
8
X
13
15
21
22
