# Supplementary material for: Structural polymorphism and population-variable coding capacity of HERV-K(HML-2) in human pangenomes
Source: bioRxiv. 2026 Sep 28:2026.09.22.753638. Preprint. [Version 1] doi: 10.64898/2026.09.22.753638 (PMC13648407; doi:10.64898/2026.09.22.753638)
Supplement: Supplementary file 2 [file media-2.zip › Supplementary_Data/Table_S14/telomeric_phylogeny/Telomeric_phylogeny.pdf]

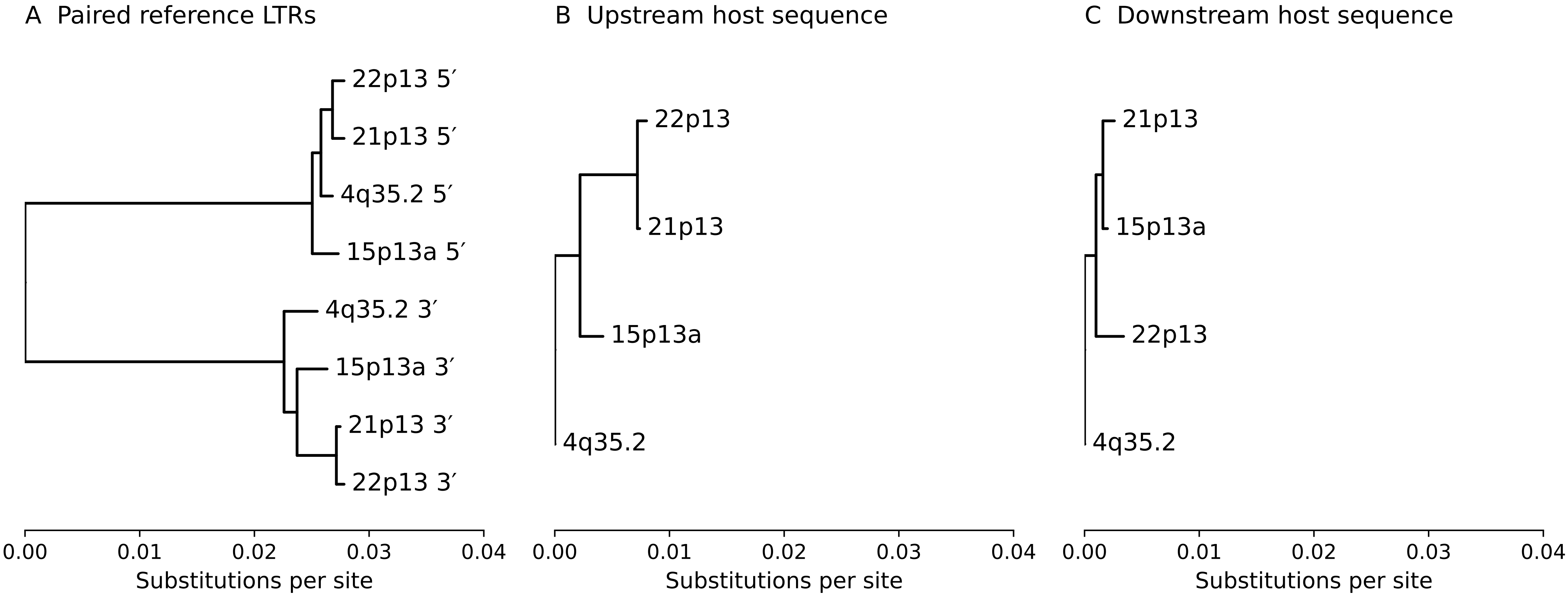
A Paired reference LTRs
22p13 5′
21p13 5′
4q35.2 5′
15p13a 5′
4q35.2 3′
15p13a 3′
21p13 3′
22p13 3′
0.00
0.01
0.02
0.03
0.04
Substitutions per site
B Upstream host sequence
22p13
21p13
15p13a
4q35.2
0.00
0.01
0.02
0.03
0.04
Substitutions per site
C Downstream host sequence
21p13
15p13a
22p13
4q35.2
0.00
0.01
0.02
0.03
0.04
Substitutions per site
