# Supplementary material for: Structural polymorphism and population-variable coding capacity of HERV-K(HML-2) in human pangenomes
Source: bioRxiv. 2026 Sep 28:2026.09.22.753638. Preprint. [Version 1] doi: 10.64898/2026.09.22.753638 (PMC13648407; doi:10.64898/2026.09.22.753638)
Supplement: Supplementary file 2 [file media-2.zip › Supplementary_Data/Table_S15/Figure_S20_array_nucleotide_variation.pdf]

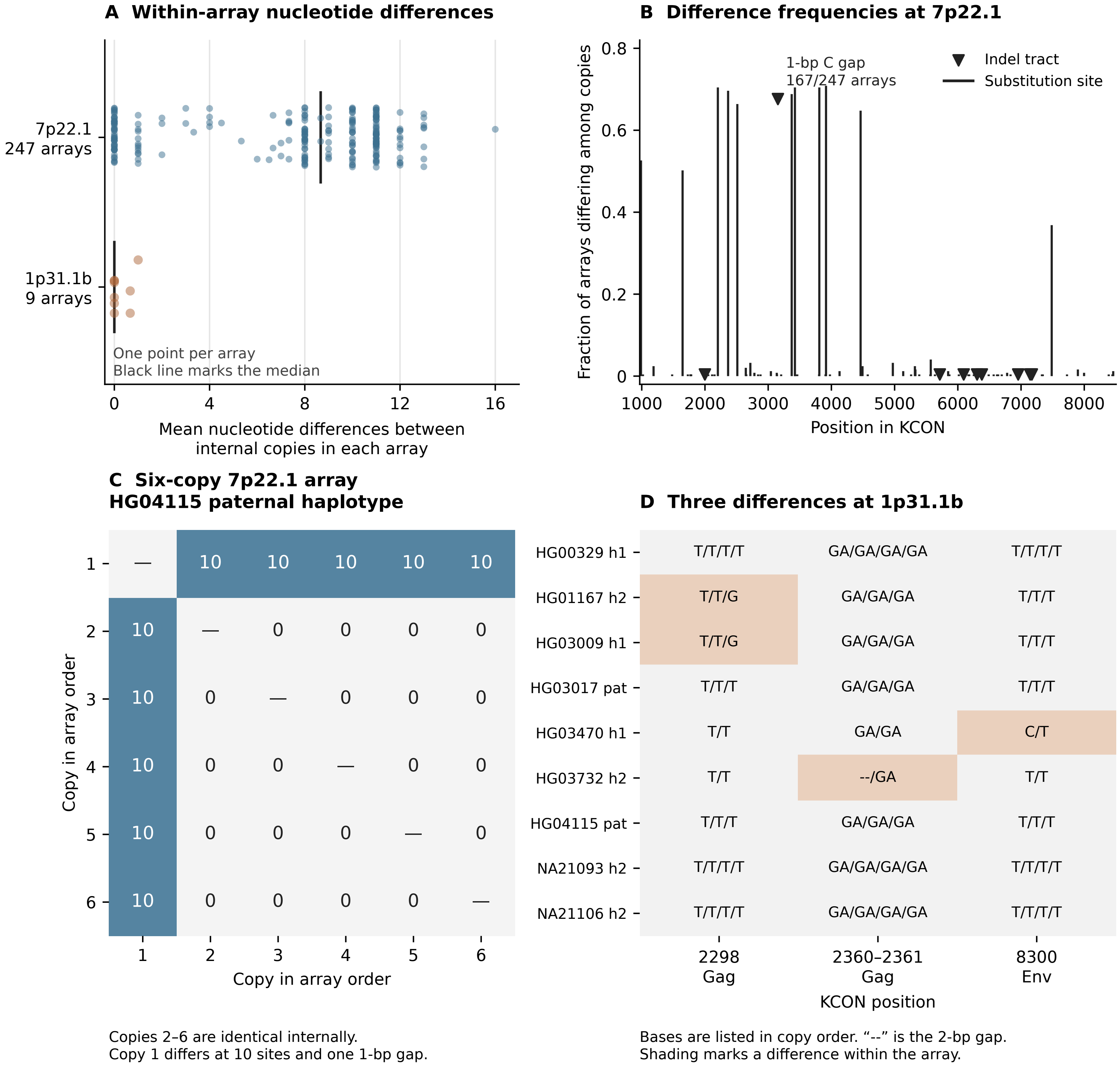
A Within-array nucleotide differences
7p22.1
247 arrays
1p31.1b
9 arrays
One point per array
Black line marks the median
0
4
8
12
16
Mean nucleotide differences between internal copies in each array
B Difference frequencies at 7p22.1
1-bp C gap
167/247 arrays
Indel tract
Substitution site
Fraction of arrays differing among copies
0.8
0.6
0.4
0.2
0
1000
2000
3000
4000
5000
6000
7000
8000
Position in KCON
C Six-copy 7p22.1 array
HG04115 paternal haplotype
1 — 10 10 10 10 10
2 10 — 0 0 0 0
3 10 0 — 0 0 0
4 10 0 0 — 0 0
5 10 0 0 0 — 0
6 10 0 0 0 0 —
1 2 3 4 5 6
Copy in array order
Copy in array order
Copies 2–6 are identical internally.
Copy 1 differs at 10 sites and one 1-bp gap.
D Three differences at 1p31.1b
HG00329 h1 T/T/T/T GA/GA/GA/GA T/T/T/T
HG01167 h2 T/T/G GA/GA/GA T/T/T
HG03009 h1 T/T/G GA/GA/GA T/T/T
HG03017 pat T/T/T GA/GA/GA T/T/T
HG03470 h1 T/T GA/GA C/T
HG03732 h2 T/T --/GA T/T
HG04115 pat T/T/T GA/GA/GA T/T/T
NA21093 h2 T/T/T/T GA/GA/GA/GA T/T/T/T
NA21106 h2 T/T/T/T GA/GA/GA/GA T/T/T/T
2298
Gag
2360–2361
Gag
8300
Env
KCON position
Bases are listed in copy order. “--” is the 2-bp gap.
Shading marks a difference within the array.
