# Supplementary material for: Structural polymorphism and population-variable coding capacity of HERV-K(HML-2) in human pangenomes
Source: bioRxiv. 2026 Sep 28:2026.09.22.753638. Preprint. [Version 1] doi: 10.64898/2026.09.22.753638 (PMC13648407; doi:10.64898/2026.09.22.753638)
Supplement: Supplementary file 2 [file media-2.zip › Supplementary_Data/Table_S17/Figure_5_type1_persistent_recombining_cassette.pdf]

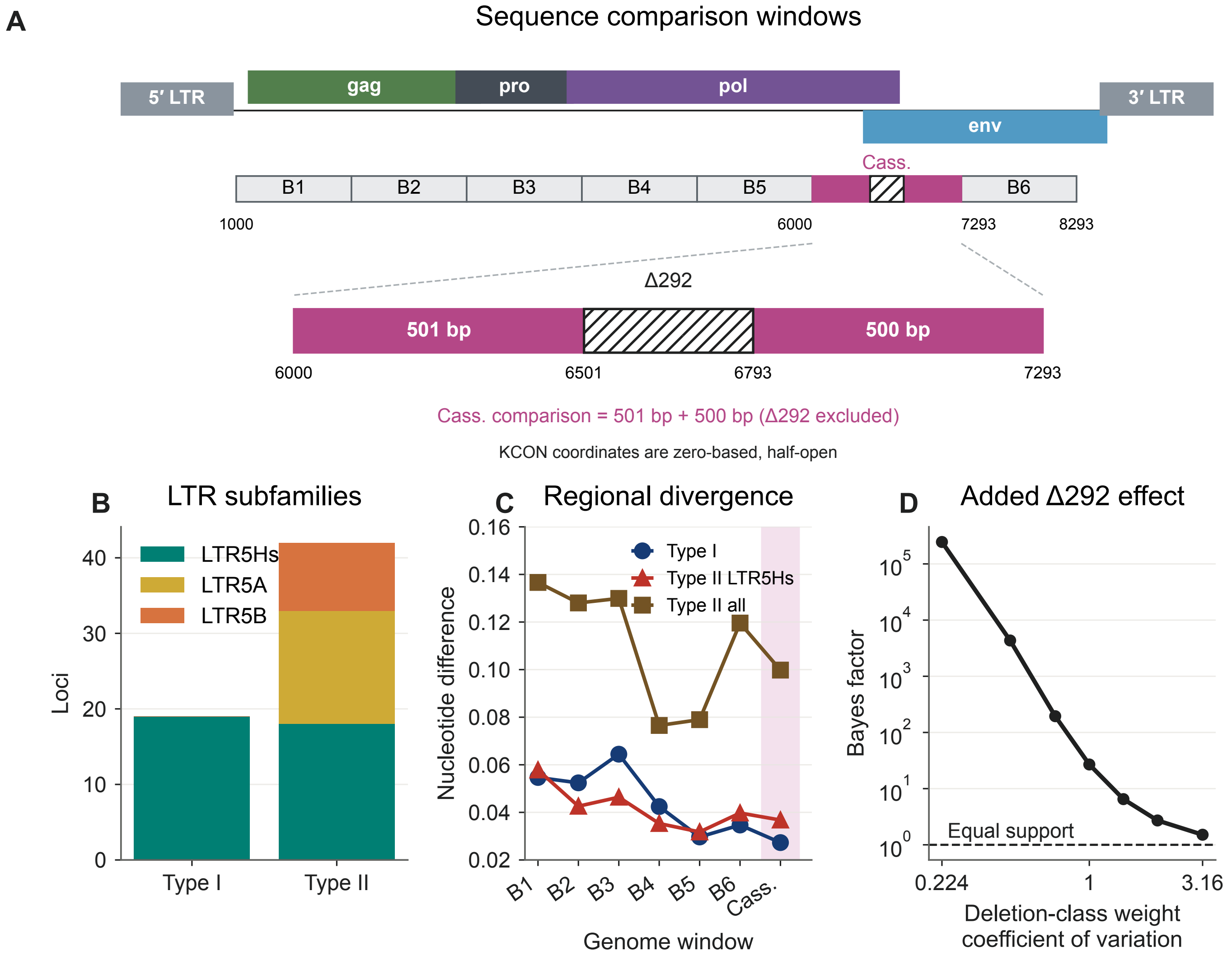
A
Sequence comparison windows
5′ LTR
gag
pro
pol
3′ LTR
env
Cass.
B1
B2
B3
B4
B5
B6
1000
6000
7293
8293
Δ292
501 bp
500 bp
6000
6501
6793
7293
Cass. comparison = 501 bp + 500 bp (Δ292 excluded)
KCON coordinates are zero-based, half-open
B
LTR subfamilies
LTR5Hs
LTR5A
LTR5B
Loci
0
10
20
30
40
Type I
Type II
C
Regional divergence
Type I
Type II LTR5Hs
Type II all
Nucleotide difference
0.02
0.04
0.06
0.08
0.10
0.12
0.14
0.16
B1
B2
B3
B4
B5
B6
Cass.
Genome window
D
Added Δ292 effect
Bayes factor
10^0
10^1
10^2
10^3
10^4
10^5
Equal support
0.224
1
3.16
Deletion-class weight
coefficient of variation
