# Supplementary material for: Structural polymorphism and population-variable coding capacity of HERV-K(HML-2) in human pangenomes
Source: bioRxiv. 2026 Sep 28:2026.09.22.753638. Preprint. [Version 1] doi: 10.64898/2026.09.22.753638 (PMC13648407; doi:10.64898/2026.09.22.753638)
Supplement: Supplementary file 2 [file media-2.zip › Supplementary_Data/Table_S17/Figure_S17_cassette_consensus_and_similarity.pdf]

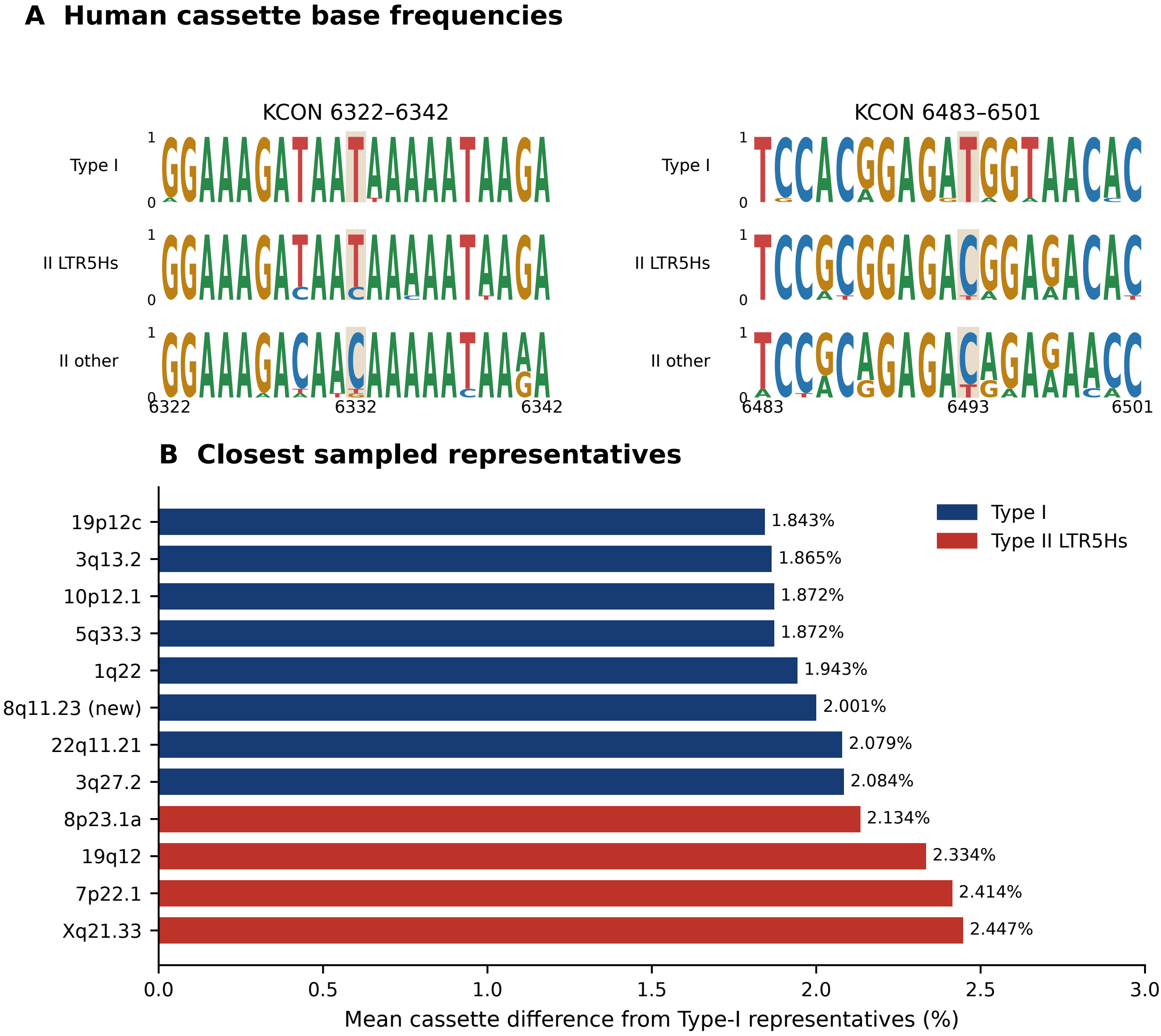
A Human cassette base frequencies
KCON 6322–6342
KCON 6483–6501
Type I
II LTR5Hs
II other
1
0
6322
6332
6342
6483
6493
6501
B Closest sampled representatives
19p12c
3q13.2
10p12.1
5q33.3
1q22
8q11.23 (new)
22q11.21
3q27.2
8p23.1a
19q12
7p22.1
Xq21.33
1.843%
1.865%
1.872%
1.872%
1.943%
2.001%
2.079%
2.084%
2.134%
2.334%
2.414%
2.447%
Type I
Type II LTR5Hs
0.0
0.5
1.0
1.5
2.0
2.5
3.0
Mean cassette difference from Type-I representatives (%)
