# Supplementary material for: Structural polymorphism and population-variable coding capacity of HERV-K(HML-2) in human pangenomes
Source: bioRxiv. 2026 Sep 28:2026.09.22.753638. Preprint. [Version 1] doi: 10.64898/2026.09.22.753638 (PMC13648407; doi:10.64898/2026.09.22.753638)
Supplement: Supplementary file 2 [file media-2.zip › Supplementary_Data/TypeI_deletion_models/figures/Figure_S12.pdf]

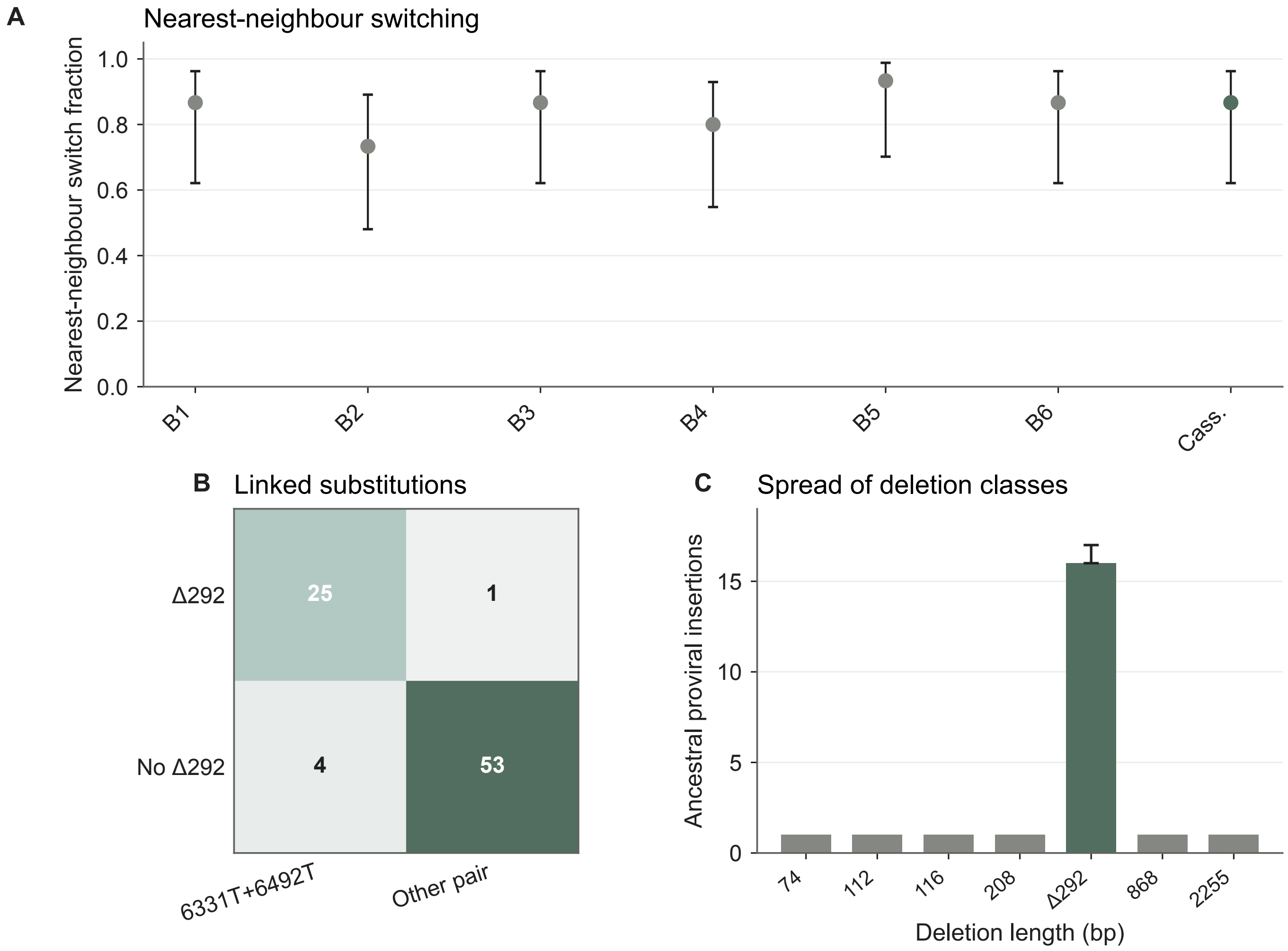
A
Nearest-neighbour switching
Nearest-neighbour switch fraction
0.0
0.2
0.4
0.6
0.8
1.0
B1
B2
B3
B4
B5
B6
Cass.
B
Linked substitutions
Δ292
No Δ292
25
1
4
53
6331T+6492T
Other pair
C
Spread of deletion classes
Ancestral proviral insertions
0
5
10
15
74
112
116
208
Δ292
868
2255
Deletion length (bp)
