# Supplementary material for: Structural polymorphism and population-variable coding capacity of HERV-K(HML-2) in human pangenomes
Source: bioRxiv. 2026 Sep 28:2026.09.22.753638. Preprint. [Version 1] doi: 10.64898/2026.09.22.753638 (PMC13648407; doi:10.64898/2026.09.22.753638)
Supplement: Supplementary file 2 [file media-2.zip › Supplementary_Data/TypeI_deletion_models/figures/Figure_S14.pdf]

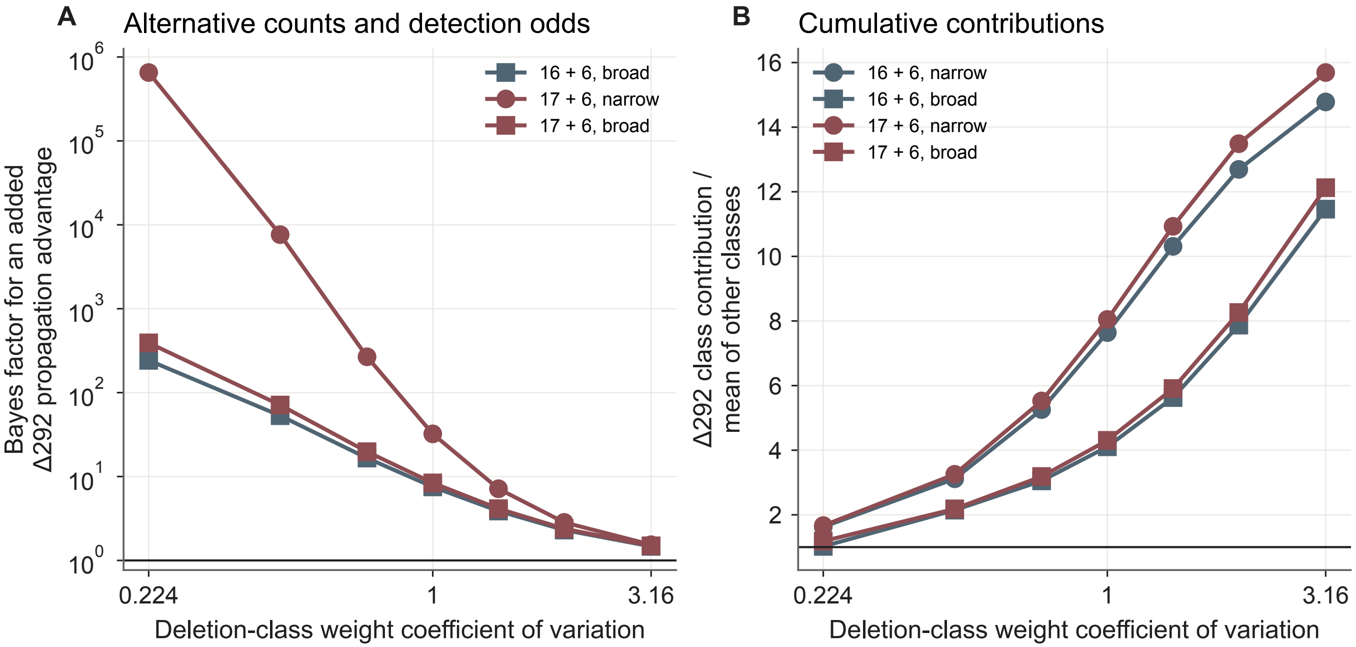

A
Alternative counts and detection odds
Bayes factor for an added Δ292 propagation advantage
10^0
10^1
10^2
10^3
10^4
10^5
10^6
16 + 6, broad
17 + 6, narrow
17 + 6, broad
0.224
1
3.16
Deletion-class weight coefficient of variation
B
Cumulative contributions
Δ292 class contribution / mean of other classes
2
4
6
8
10
12
14
16
16 + 6, narrow
16 + 6, broad
17 + 6, narrow
17 + 6, broad
0.224
1
3.16
Deletion-class weight coefficient of variation
