# Supplementary material for: Structural polymorphism and population-variable coding capacity of HERV-K(HML-2) in human pangenomes
Source: bioRxiv. 2026 Sep 28:2026.09.22.753638. Preprint. [Version 1] doi: 10.64898/2026.09.22.753638 (PMC13648407; doi:10.64898/2026.09.22.753638)
Supplement: Supplementary file 2 [file media-2.zip › Supplementary_Data/TypeI_deletion_models/figures/Figure_S17.pdf]

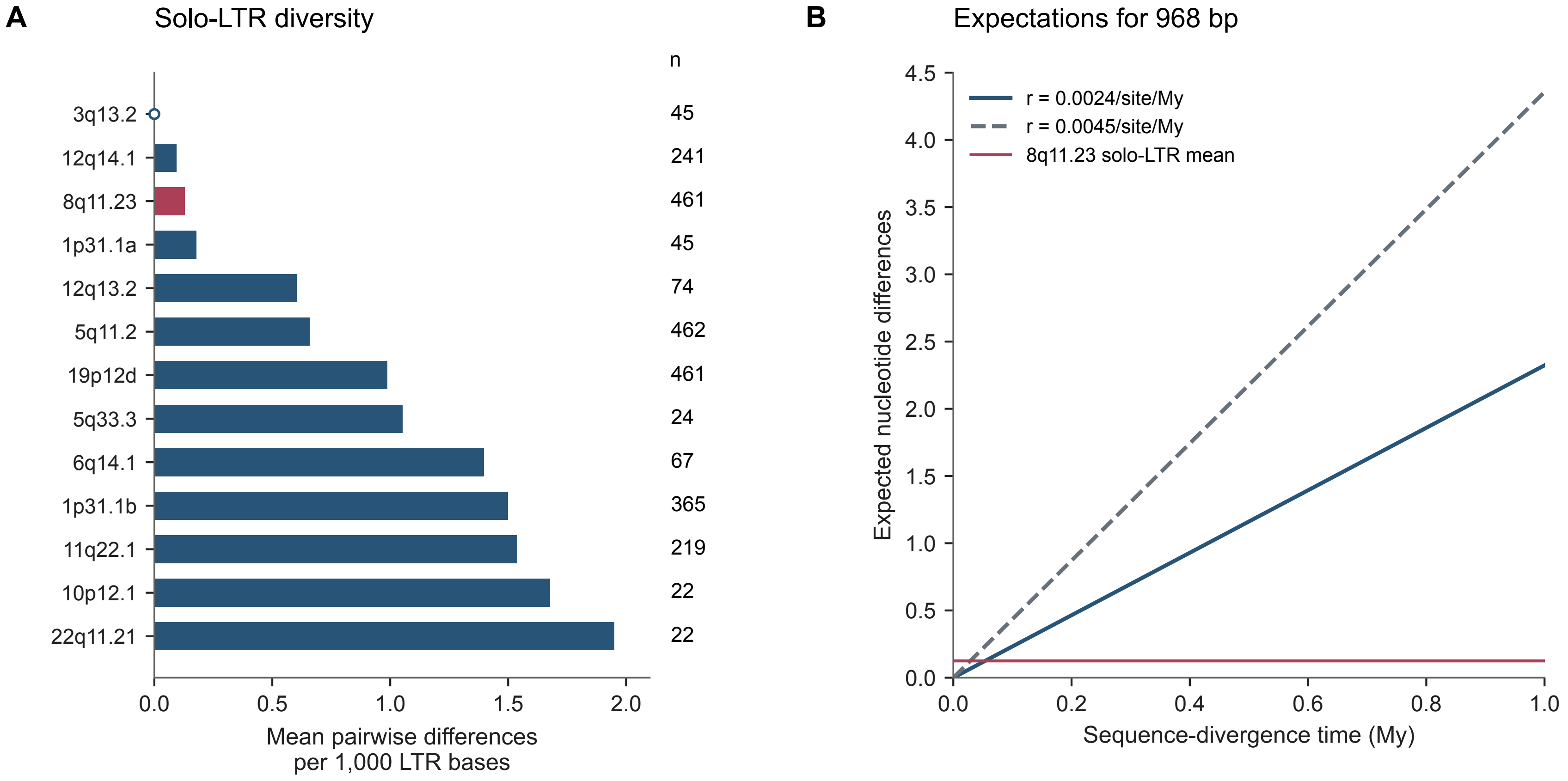
A
Solo-LTR diversity
n
3q13.2
12q14.1
8q11.23
1p31.1a
12q13.2
5q11.2
19p12d
5q33.3
6q14.1
1p31.1b
11q22.1
10p12.1
22q11.21
45
241
461
45
74
462
461
24
67
365
219
22
22
0.0
0.5
1.0
1.5
2.0
Mean pairwise differences
per 1,000 LTR bases
B
Expectations for 968 bp
r = 0.0024/site/My
r = 0.0045/site/My
8q11.23 solo-LTR mean
Expected nucleotide differences
0.0
0.5
1.0
1.5
2.0
2.5
3.0
3.5
4.0
4.5
0.0
0.2
0.4
0.6
0.8
1.0
Sequence-divergence time (My)
